# System dynamics modelling of health workforce planning to address future challenges of Thailand’s Universal Health Coverage

**DOI:** 10.1186/s12960-021-00572-5

**Published:** 2021-03-10

**Authors:** Borwornsom Leerapan, Pard Teekasap, Nipaporn Urwannachotima, Wararat Jaichuen, Kwanpracha Chiangchaisakulthai, Khunjira Udomaksorn, Aronrag Meeyai, Thinakorn Noree, Krisada Sawaengdee

**Affiliations:** 1 Faculty of Medicine Ramathibodi Hospital, Mahidol University, 270 Rama VI Road, Ratchathewi, Bangkok, Thailand; 2 Faculty of Business Administration, Stamford International University, Bangkok, Thailand; 3 Faculty of Dentistry, Chulalongkorn University, Bangkok, Thailand; 4 International Health Policy Program, Thailand (IHPP), Ministry of Public Health, Nonthaburi, Thailand; 5 Office of the Permanent Secretary, Ministry of Public Health, Nonthaburi, Thailand; 6 Faculty of Pharmacy, Prince of Songkla University, Songkla, Thailand; 7 Faculty of Public Health, Mahidol University, Bangkok, Thailand; 8 Oxford Centre for Global Health Research, Nuffield Department of Medicine, University of Oxford, Oxford, UK

**Keywords:** Human resource for health, Health workforce, Strategic planning, Care delivery models, Health systems performance, Group model building, Causal loop diagram, System dynamic modelling

## Abstract

**Background:**

System dynamics (SD) modelling can inform policy decisions under Thailand's Universal Health Coverage. We report on this thinking approach to Thailand's strategic health workforce planning for the next 20 years (2018–2037).

**Methods:**

A series of group model building (GMB) sessions involving 110 participants from multi-sectors of Thailand's health systems was conducted in 2017 and 2018. We facilitated policymakers, administrators, practitioners and other stakeholders to co-create a causal loop diagram (CLD) representing a shared understanding of why the health workforce's demands and supplies in Thailand were mismatched. A stock and flow diagram (SFD) was also co-created for testing the consequences of policy options by simulation modelling.

**Results:**

The simulation modelling found hospital utilisation created a vicious cycle of constantly increasing demands for hospital care and a constant shortage of healthcare providers. Moreover, hospital care was not designed for effectively dealing with the future demands of ageing populations and prevalent chronic illness. Hence, shifting emphasis to professions that can provide primary care, intermediate care, long-term care, palliative care, and end-of-life care can be more effective.

**Conclusions:**

Our SD modelling confirmed that shifting the care models to address the changing health demands can be a high-leverage policy of health workforce planning, although very difficult to implement in the short term.

## Background

Thailand achieved Universal Health Coverage (UHC) in 2002 after decades of healthcare infrastructure development and experimenting with several financial risk protection schemes [1]. Since then, every Thai citizen was covered under one of the three major health financing schemes. Even before implementing Thai UHC, the planning of Thailand’s health workforce has been incorporated into the National Economic and Social Development Plans. Over the decades, public healthcare facilities have been expanded nationwide. Thailand successfully built provincial hospitals in every province of Thailand by 1976, followed by the development of community hospitals in every district by 1991, and the modernization of primary care centres at the sub-district level during the 1990s [2]. The Twelfth National Economic and Social Development Plan (2017–2021) calls for “Preparation of the Workforce and Capacity Enhancement of People of All Ages” by promoting a healthy population and encouraging healthy behaviour and reducing environmental risks that could harm people’s lives, and “Creating a Just Society and Reducing Inequality” by an emphasis on the quality of education and healthcare for the disadvantaged and those living in remote areas [3]. Nonetheless, historically, the national plans of workforce production and development also have focused on the providers in the public sector.

Health workforce or human resources for health (HRH) is one the building blocks of health systems, and the types and the number of healthcare providers needed in each health system are closely linked to how healthcare is organized in each country [4, 5]. Like many other countries, healthcare systems in Thailand have been organized since the last century to make them more responsive to acute illness. Hospitals, which are historically designed for acute care, are currently the dominant providers for the UHC beneficiaries. Policymakers of the three publicly-financed health funds have allocated most resources to providers in public hospital settings, but not in others providing in primary care, intermediate care, long-term care, palliative care, and end-of-life care. As a result, hospitals have been the major employers of healthcare providers in Thailand thus far.

More recently, a rapidly increased prevalence of non-communicable diseases and aging populations, as well as insufficient facilities specifically designed for chronic and elderly care, have limited the effectiveness of Thailand’s first decade of the Universal Coverage Scheme (UCS) [6], the largest public healthcare financing scheme under Thailand’s UHC. This rapid change of the population’s health demands could also aggravate the complex problem of inadequate health workforce domestically, which eventually can lead to equitable access to quality healthcare under Thai UHC. The expansion of private healthcare facilities in the private sector since the early 2000s also created a domestic “brain drain” of the health workforce, especially physicians, despite the innovative policies to retain them in the public sector.

Although Thailand has produced more healthcare workers every year, with the number of physicians or nurses per capita has been rapidly increased over the decades, many Thai UHC beneficiaries still have limited access to quality healthcare. Research has shown that public hospitals in Thailand, given fixed inputs, have produced services relatively close to their capacity [7]. The long waiting time of patients at the outpatient department of every public hospital nationwide is self-evident for the current mismatches between supplies and demands of the health workforce in Thailand. Therefore, increasing the number of health workforce produced each year will only get us thus far. This protracted problems of insufficient quantity of the health workforce in Thailand is complex. Policymakers would require a comprehensive analysis and decision support tools with a systems thinking approach to not only address all possible causes, but also to identify the high-leverage points in health systems to alleviate such problems. Therefore, a more comprehensive strategic planning that includes the reforms of healthcare delivery itself is needed to address this complex problem.

We aimed to analyse what causes the chronic mismatches of supply and demands for the health workforce in Thailand and to synthesise more sustainable solutions to supply population health demands in Thailand in the future adequately. Using a systems thinking approach and a structured process of group model building (GMB) [8], we engaged with stakeholders who are embedded in a system to examine the nature of these complex problems, the pattern of system behaviours over time, highlight the feedbacks within the systems, and constructed a system dynamics (SD) modelling of health workforce planning to address future challenges of Thailand’s UHC. In the present study, we report on developing a whole-systems perspective of problems related to the health workforce in Thailand in the next 20 years, what causes them, and how potential systems interventions can be identified and tested by our simulation model.

## Methods

### Setting

The study was carried out as a collaborative project by Mahidol University’s Faculty of Medicine Ramathibodi Hospital and Thailand’s Ministry of Public Health (MoPH). A series of group model building (GMB) sessions were conducted in our workshops held in Bangkok and Nonthaburi, Thailand, under the authorities of MoPH during 2017 and 2018.

### Study design and participants

The present study employed systems thinking and modelling methodology based on the system dynamics approach [9]. We used system dynamics (SD) as our mathematical modelling method that employs systems thinking tools to understand complex systems' behaviours over time [10]. SD is among the most popular modelling methods in health policy and healthcare research [11, 12]. But unlike agent-based models that aim to capture micro-level system behaviours (i.e. human decision-making and heterogeneous interactions between individuals), SD models address macro-level system behaviours (i.e. changes or movement of resources in complex systems over time) [13]. Using differential equations to model changing variables over a period of time while allowing for feedback and various interactions and delays, SD models also address the issues of simultaneity or the mutual causation of systems behaviours [10]. While SD models may ignore fine details of complex health systems, especially actions of individual patients or healthcare providers, the method allows for the model breadth to explore long-term effects of strategic changes in our complex health systems. We also adopted five major phases of the systems thinking and modelling methodology put forth by Maani and Cavana [14], including (1) problem structuring, (2) casual loop modelling, (3) dynamic modelling, (4) scenario planning and modelling, (5) implementation and organizational learnings.

We purposefully identified the MoPH policymakers in charge of planning healthcare services and health workforce at the national levels, administrators of healthcare organizations, and healthcare practitioners from both public and private sectors as the participants of our study. Health systems researchers with an expertise in health workforce planning and healthcare labour market, educators in health professional schools within universities, and the representatives from professional councils regulating the licensing of health workforce in Thailand were also invited to participate. A total of 110 stakeholders from multi-sectors in Thai health systems participated in a series of our modelling sessions. As the stakeholders of national health workforce planning, they were facilitated to co-create a causal model that can explain the mismatches between demands and supplies of the health workforce in Thailand, which progressed from structuring the problems by connecting relevant concepts to constructing a qualitative causal loop diagrams to quantitative stock and flow diagrams for dynamic modelling and scenario planning.

### Group model building

Using “scripts” from system dynamics literature [15, 16], we facilitated the stakeholders by using a structured group model building [8, 15] to engage with our stakeholders. A series of GMB sessions were conducted in 2017 and 2018. Three facilitators trained in GMB (BL, PT, NU) led the GMB sessions. All facilitators were introduced to the customary practices of health workforce planning at the national level by the MoPH officers to understand the necessary process of Thailand’s health workforce planning before running the GMB sessions. Facilitators held four series of GMB workshops over 12 months, with an average of 40 participants attended each GMB session, and a total of 110 stakeholders participated in all sessions.

First, the facilitators and the participants discussed and agreed upon the expected outcomes in the next 20 years of the health workforce planning, and drawn the reference mode of such outcomes. We proposed a seed question: “What factors have led to an insufficiency of the health workforce in Thailand?” Then the participants generated a shared list of these factors, nominated variables, and added causal links among those variables. Then we co-created a causal loop diagram (CLD) to gain a mutual understanding of what factors caused undesirable consequences, particularly mismatches of supply and demands for the health workforce in Thailand over the decades. Second, we worked with stakeholders to gain more significant insights from the CLD. This sequence focused on the seeding question: “What are the factors that help or hurt our ability to produce and maintain an insufficient number of the health workforce in Thailand over time? The facilitators updated the raw CLDs through an iterative process during the GMB sessions, with updated causal maps presented to the participants for critique and revised in each session. Third, we turned our insights from the updated CLD into a stock and flow diagram (SFD) for SD simulation modelling. We presented the draft structure of SFD to the participants and asked for their feedback. We consulted the participants about which database is the most appropriate for extracting the parameters needed for our quantitative modelling, and what value of each parameter is. To create policy options, we also asked: “What the high-leverage points within our health systems can lead to a sufficient health workforce?” Lastly, we used our SD modelling to simulate the selected health systems outcomes for the next two decades (2018–2037) and analysed the consequences of such policy options. Before the end of our study, we presented the results of both the qualitative model (CLD) and the quantitative simulation modelling (SD modelling with scenarios planning) to the high-level executives in the Ministry of Public Health for eliciting comments and feedbacks.

Our GMB sessions produced a CLD that represents a common understanding among participating stakeholders. The critical variables discussed in the GMB sessions include the population structure of aging society, the unmet health needs of the population, utilization of healthcare services in hospital settings, utilization of healthcare services in non-hospital settings, size of the labour market of hospital care, size of the labour market in non-hospital care, people’s health literacy and self-care, and the effectiveness of population health interventions.

**Fig. 1:**
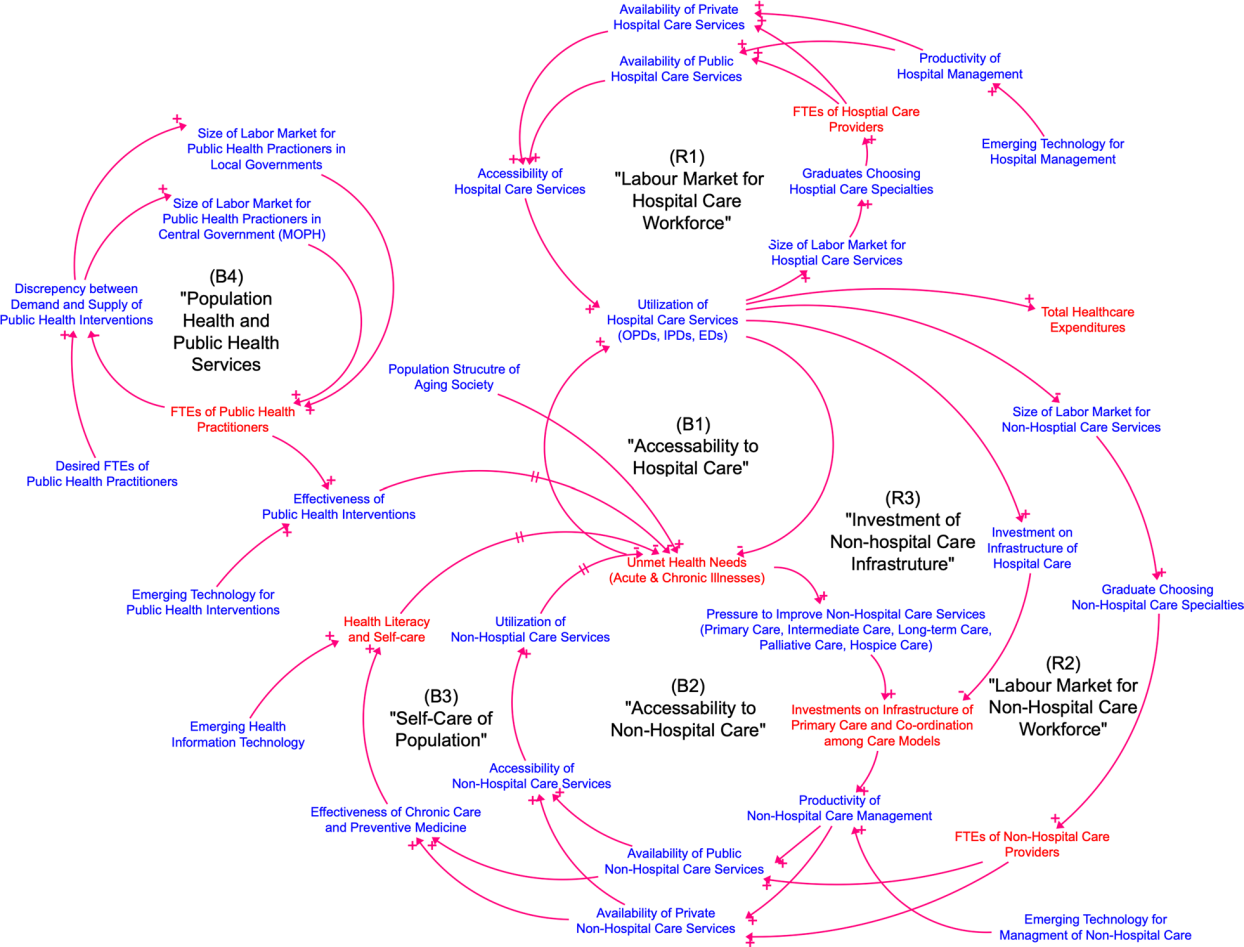
The CLD of insufficiency of the health workforce in the hospital care and the non-hospital care settings of Thailand from the GMB process

As shown in Fig. 1, the balancing and reinforcing loops constitute the dynamic hypotheses of how health system components interact and result in a steady level of unmet health needs and rising demands for utilization of hospital care. Also, increasing demands for the workforce in hospital settings leads to decreasing supplies for the workforce in non-hospital settings, medical errors, rising healthcare expenditures, and an undesirable level of population health status over time. The revised and final CLD contains seven interacting feedback loops can be categorized into the four domains, namely: (1) the relationship between hospital care utilization and the labour market for hospital care (B1 and R1); (2) the relationship between non-hospital care utilization and the labour market for non-hospital care (B2 and R2); (3) the investment on non-hospital care infrastructure (R3); and (4) public health services and drivers of population health (B3 and B4).

### Model structure

The dynamic hypotheses, as depicted on CLD, formed a basis for our development of SFD and the structure of our SD model. We constructed three modules to represent our insights from the CLD, which include factors and relationships that can lead to mismatches of supplies and demands for the health workforce in Thailand’s health systems, including (1) population module; (2) healthcare delivery module; (3) education and labour market module.

**Fig. 2:**
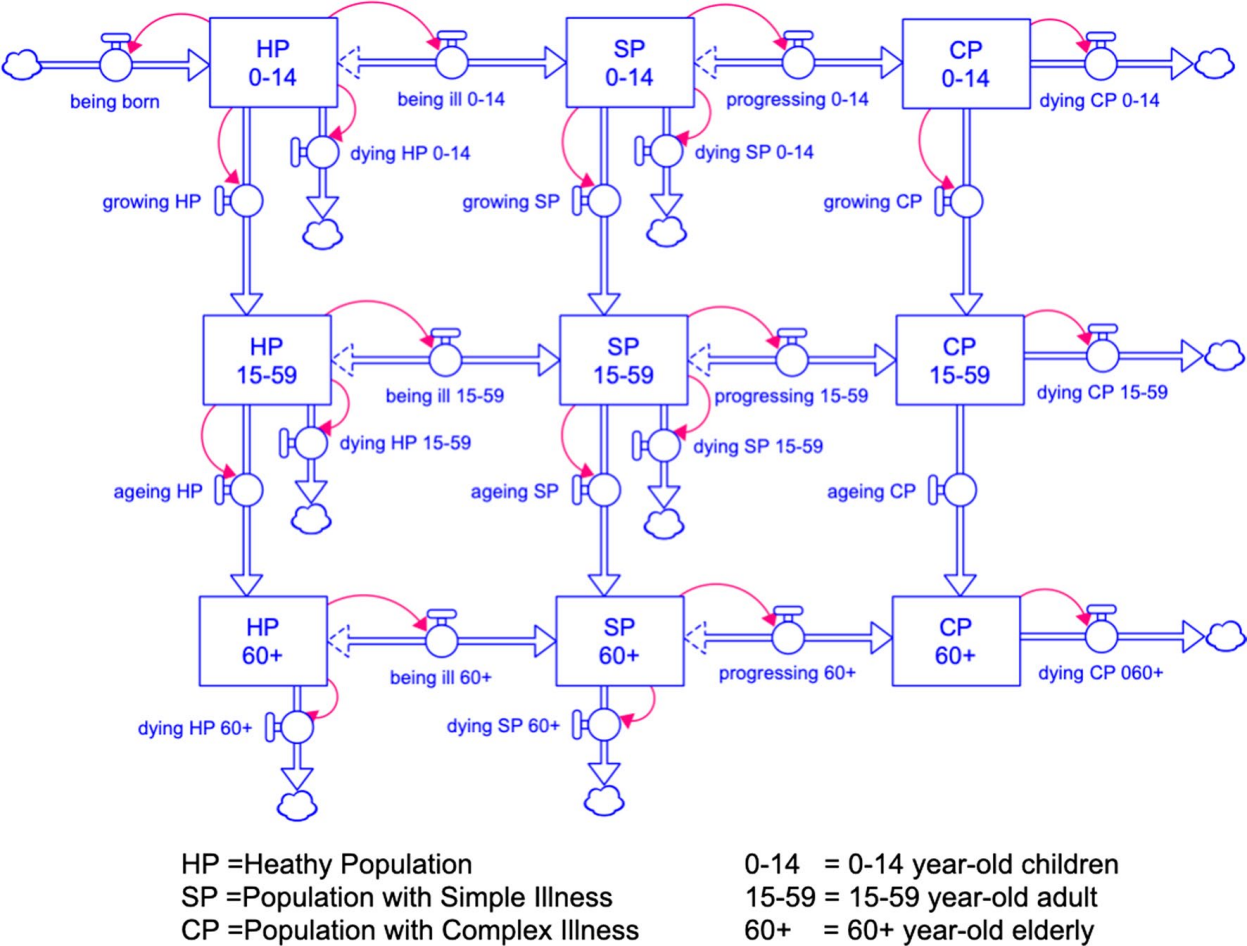
Structure of the population module showing the health states of Thai populations (healthy, with simple illnesses, with complex illnesses) and the age of Thai populations (0–14, 15–59, 60 and above)

**Fig. 3:**
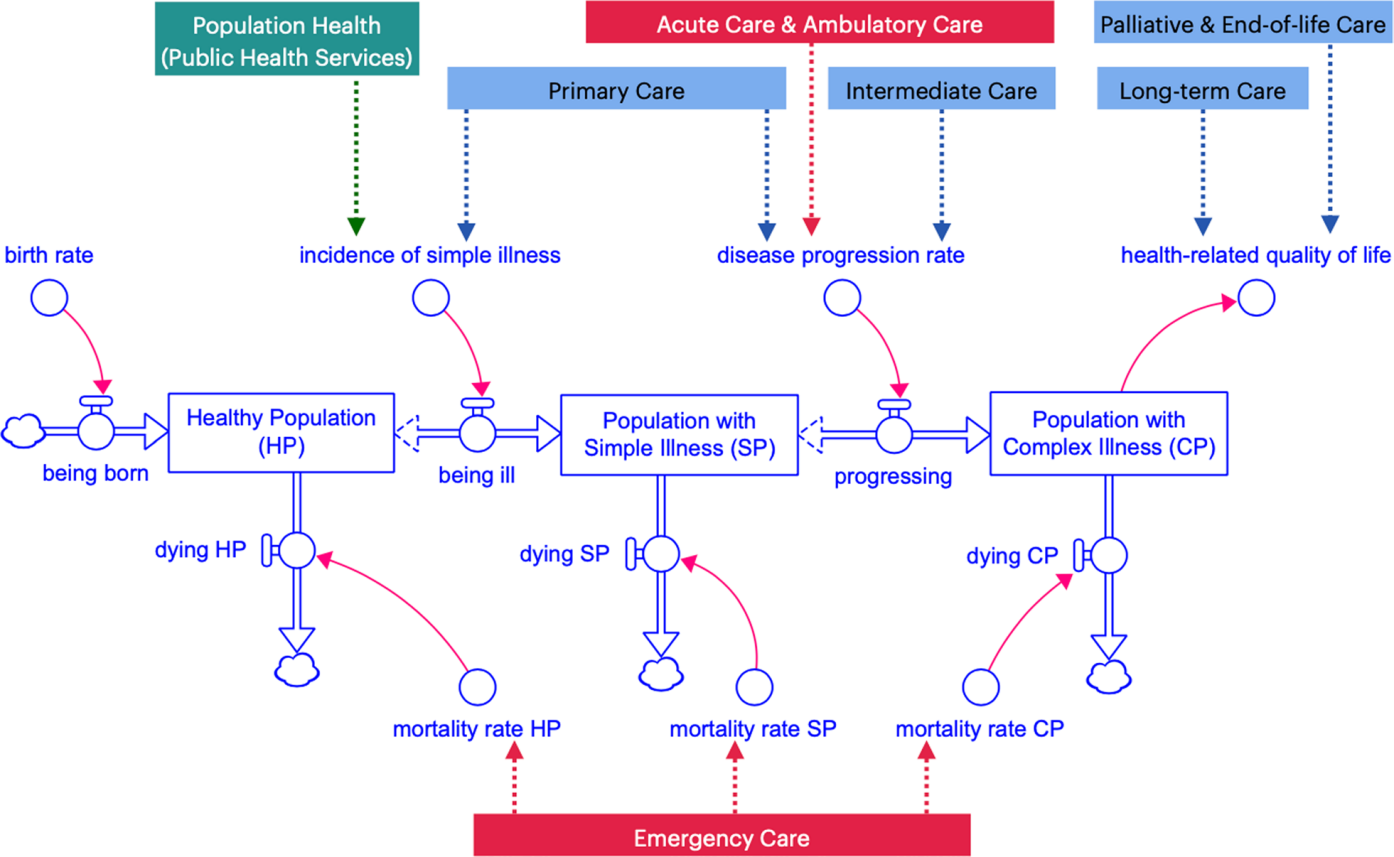
Structure of the healthcare market and its relationship with the changing population

**Fig. 4:**
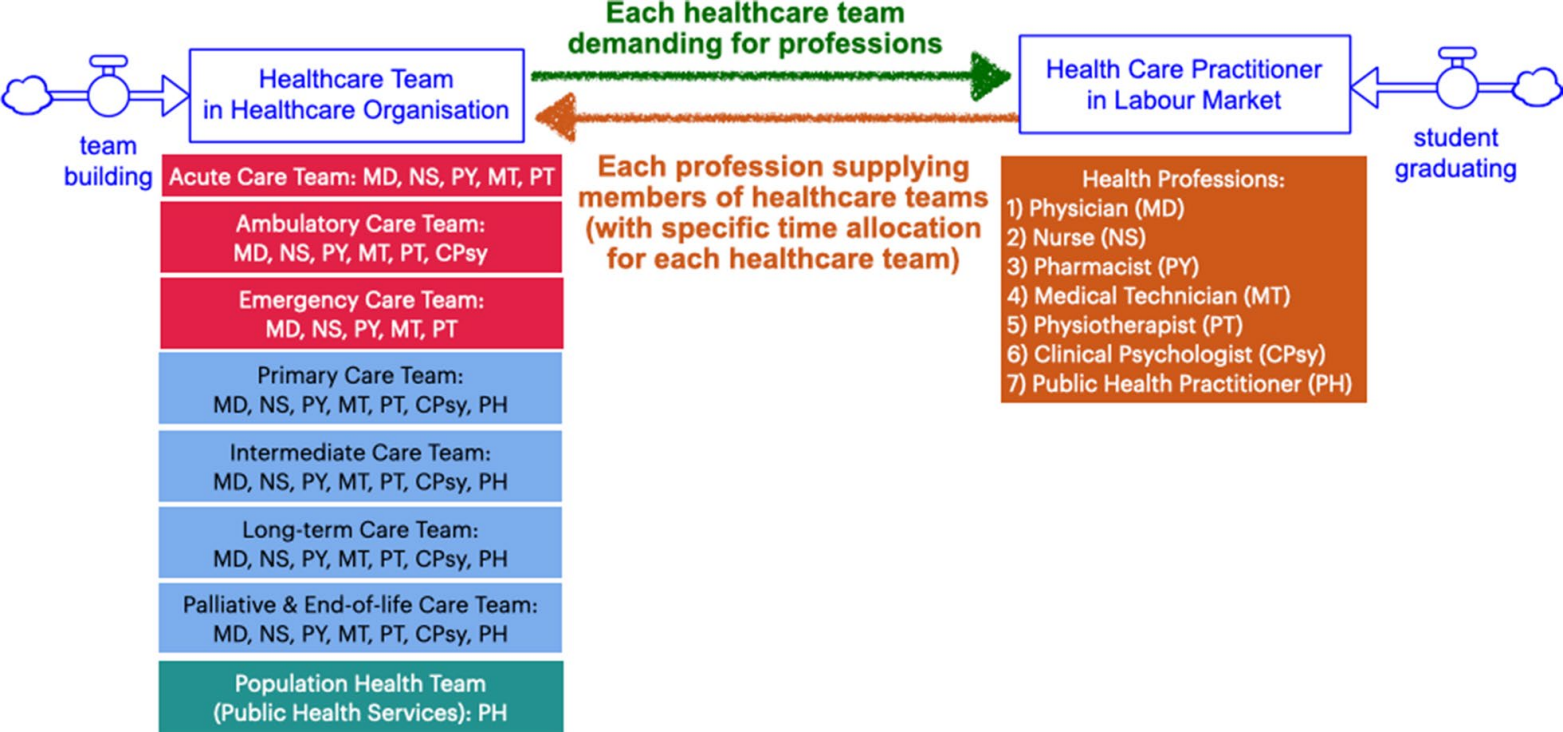
Structure of the health labour market and its relationship with the healthcare team in different healthcare models (*MD* physician/medical doctor, *NS* nurse, *PY* pharmacist, *MT* medical technologist or medical laboratory technologist, *PT* physical therapist or physiotherapist, *PH* public health practitioner/officer, *CPsy* clinical psychologist)

1. Population module:
  Considering how sufficiency of the health workforce can impact the population health status, we considered each person can occupy a health state by the levels of severity of their illness. We broke down population’s health status into three stocks: (1) healthy population (HP); (2) population with simple illnesses (SP); and (3) population with complex illnesses (CP). Each health state corresponds to the nature of patient care teams and healthcare models that would be expected to inhibit progression into or regression from more severe health states, as represented by the inflows and outflows. Each person can also progress in terms of aging. Still, we categorized the population to only three groups by ages (0–14, 15–49, 50, and above), and also corresponds to the nature of patient care teams and healthcare models usually needed in that age group. The structure of the population is depicted on Fig. 2.
2. Healthcare delivery and healthcare market module:
  In this module, we displayed the population health demands by health needs as professionally defined [17]. Hence, on the demand side of the healthcare market, each of the health states (HP, SP, CP) creates specific health demands for the health workforce and patient care teams in healthcare market, also demonstrated in Fig. 3. The accessibility and utilization of each healthcare delivery model on the population model are also described in Table 1.
  The supply side of healthcare market is determined by the health workforce's capacity within health care teams. We considered nine types of teams available in our health systems: eight patient care teams in eight care delivery models, and one public health services team. Each type of teams requires a different combination of healthcare professionals.
  The first three categories of health professional teams provide healthcare services necessarily delivered in the hospitals, including: (1) acute care teams (for inpatients), (2) ambulatory care teams (for outpatients), (3) emergency care teams (for patients with emergency injuries and illnesses), and in this study we collectively defined them as “hospital care teams”. Next, we also defined the “non-hospital care teams” as the providers of healthcare services not necessarily delivered in the hospitals, including: (4) primary care teams (for all populations), (5) intermediate care or subacute care teams (for patients who require rehabilitation), (6) long-term care teams (for the elderly and people with disabilities), (7) palliative care and end-of-life care teams (for patients with critical illnesses), and (8) dental care teams (for all populations concerned with oral health problems). For health workforce whose work is not a direct care for individual patients but population-based practices, such as community-based projects of disease prevention and health promotion, we considered them a part of (9) population health or public health services teams.
  In the present study, we excluded the dental care from our model because of its different nature of health demands and a separate group of healthcare providers who serve such needs, namely dentists and dental auxiliaries. Another SD model was constructed in a separated study of dental workforce planning.
3. Healthcare education and labour market module:
  The structure of the health labour market and its relationship with health workforce education and training are shown in Fig. 4. The composition of health professions that forms a typical membership of each healthcare model is also shown in Fig. 4. The supply side of the healthcare market is also the demand side of this healthcare labour market. Hence, the demands for hiring the health workforce in each profession are also determined by the capacity of the health workforce in health care teams and population health team. Each team demanding for a different combination of professions. At the same time, each profession entering the health labour market also supplies the members of healthcare teams, with specific time allocation or full-time equivalent (FTE) for each team as listed on Table 2.

**Table 1:** Effectiveness of utilization of each healthcare delivery model on the population module

Models of care	Users	Effects
1. Acute care (IPD)	Population with complex illnesses of all ages	Decrease mortality rate Increase regression from CP to SP
2. Ambulatory care	Population with complex illnesses of all ages	Increase regression from CP to SP
3. Emergency care	All population groups	Decrease mortality rate
4. Primary care	A healthy population of all ages Population with simple illnesses of all ages Population with complex illnesses of all ages	Decrease progression from HP to SP Decrease progression from SP to CP Increase the health-related quality of life (HRQoL) in CP users
5. Palliative care	Population with complex illnesses of all ages	No effects on health status Positive impacts on quality of life (CP)
6. Long-term care	Elderly (population with simple illnesses, population with complex illnesses) Disabilities (young and adult) Excluding a healthy population of all ages	No effects on health status Positive impacts on quality of life (CP)
7. Intermediate care	Population with complex illnesses of all age	Increase regression from CP to SP
8. Population health	A healthy population of all ages	Decrease incidence via environmental and behavioral changes

**Table 2:** Model parameters used in the simulation model

Model parameter	Unit	Parameter initial value (2015)	Source
*Population module*			
Healthy population (HP)	Person	9,307,085 (0–14), 33,319,965 (15–59), 3,661,054 (60+)	The 2015 Health And Welfare Survey, Thailand National Statistical Office
Population with simple illnesses (SP)	Person	2,283,981 (0–14), 9,478,869 (15–59), 5,951,453 (60+)	The 2015 Health And Welfare Survey, Thailand National Statistical Office
Population with complex illnesses (CP)	Person	414,449 (0–14), 2,029,071 (15–59), 717,808 (60+)	The 2015 Health And Welfare Survey, Thailand National Statistical Office
Birth ratio	Per year	0.0127	Institute for Population and Social Research, Mahidol University
Death ratio	Per year	0.00113 (0–14, HP), 0.00113 (0–14, SP), 0.00226 (0–14, CP), 0.00285 (15–59, HP), 0.00285 (15–59, SP), 0.0057 (15–59, CP), 0.02844 (60+, HP), 0.02844 (60+, SP), 0.05688 (60+, CP)	Burden of Disease (BOD), IHPP Thailand and Model validation by the authors
Progression ratio	Per year	0.05 (0–14), 0.05 (15–59), 0.06 (60+)	Expert opinions from GMB sessions and Model validation by the authors
Curing effect of care	Dimensionless	0.5 (acute care to CP, 0–14), 0.4 (ambulatory care to CP, 0–14), 0.5 (acute care to CP, 15–59), 0.35 (ambulatory care to CP, 15–59), 0.2 (acute care to CP, 60+), 0.05 (ambulatory care to CP, 60+), 0.5 (from SP to HP, 0–14), 0.5 (from SP to HP, 15–59), 0.25 (from SP to HP, 60+)	Expert opinions from GMB sessions and Model validation by the authors
Health-related quality of life (HRQoL)	Dimensionless	1 (HP), 0.8 (SP), 0.75 (CP with care access), 0.5 (CP without care access)	Expert opinions from GMB sessions
Treatment duration	Year	1	Expert opinions from GMB sessions
Incidence ratio adjustment from access to healthcare	Dimensionless	1–1.2	Expert opinions from GMB sessions
Incidence ratio adjustment from health literacy	Dimensionless	0.5–1	Expert opinions from GMB sessions
*Healthcare module*			
Actual position (public sector)	Person	17,892 (MD, MoPH), 106,300 (NS, MoPH), 8449 (PY, MoPH), 3761 (MT, MoPH), 2502 (PT, MoPH), 33,300 (PH, MoPH), 212 (CPsy, MoPH), 8891 (MD, other public), 19,923 (NS, other public), 1024 (PY, other public), 781 (MT, other public), 309 (PT, other public), 41 (PH, other public), 49 (CPsy, other public)	MoPH and HRH survey by the authors
Actual position (private sector)		14,641 (MD), 18,444 (NS), 642 (PY), 500 (MT), 828 (PT), 0 (PH), 0 (CPsy)	MoPH and HRH survey by the authors
Actual position (local government)		911 (MD), 4660 (NS), 258 (PY), 87 (MT), 109 (PT), 183 (PH), 74 (CPsy)	MoPH and HRH survey by the authors
Demand for healthcare	Episode/person/year	2 (primary care, 0–14. HP), 2 (primary care, 15–59, HP), 2 (primary care, 60+, HP), 9 (primary care, 0–14, SP), 6 (primary care, 15–59, SP), 12 (primary care, 60+, SP), 9 (primary care, 0–14, SP), 6 (primary care, 15–59, SO), 12 (ambulatory care, 0–14, CP), 3 (ambulatory care, 15–59, CP), 12 (ambulatory care, 60+, CP), 3 (acute care, all ages, CP), 1 (emergency care, all ages, CP), 40 (intermediate care, all ages, CP), 40 (palliative care, all ages, CP), 12 (long-term care, all ages, CP)	Expert opinions from GMB sessions
Cost per service (public sector)	Baht/episode	740 (primary care), 7583 (acute care), 2000 (emergency care), 780 (ambulatory care), 1211 (intermediate care), 2000 (palliative care), 3840 (long-term care)	Chiangchaisakultha et al. [31], and Expert opinions from GMB sessions
Cost per service (private sector)	Baht/episode	15,000 (primary care), 84,734 (acute care), 8000 (emergency care), 3480 (ambulatory care), 2500 (intermediate care), 4000 (palliative care), 8000 (long-term care)	Pongpattracha et al. [32] and Expert opinions from GMB sessions
HRH production cost per head	Baht/person	2,340,000 (MD), 600,000 (PY), 480,000 (NS, MT, PT, PH, CPSY)	Praboromarajchanok for Health Workforce Development, MoPH
Labor cost per head	Baht/person/per year	1,060,140 (MD), 463,716 (NS), 501,408 (PY), 382,548 (MT), 283,128 (PT), 295,440 (CPSY)	Chiangchaisakultha et al. [33] and Expert opinions from GMB sessions
The targeted number of public health officers	Person	50,000	Expert opinions from GMB sessions (estimated from the desirable ratio of public health practitioner per population of 1:1250)
Healthcare team (public sector—central government)	Team	4150 (primary care, MoPH), 0 (primary care, other public), 3345 (acute care, MoPH), 499 (acute care, other public), 1280 (emergency care, MoPH), 398 (emergency care, other public), 292 (intermediate care, MoPH), 94 (intermediate care, other public), 292 (intermediate care, MoPH), 128 (palliative care, MoPH), 38 (palliative care, other public), 4278 (long-term care, MoPH), 0 (long-term care, other public), 3741 (ambulatory care, MoPH), 616 (ambulatory care, other public)	The Permanent Secretary Office, MoPH and Expert opinions from GMB sessions
Healthcare team (public sector—local government)	Team	76 (primary care), 70 (acute care), 37 (emergency care), 33 (intermediate care), 0 (palliative care), 16 (long-term care), 158 (ambulatory care)	The Permanent Secretary Office, MoPH and Expert opinions from GMB sessions
Healthcare team (private sector)	Team	100 (primary care), 2153 (acute care), 323 (emergency care), 94 (intermediate care), 10 (palliative care), 0 (long-term care), 3367 (ambulatory care)	The Permanent Secretary Office, MoPH and Expert opinions from GMB sessions
Practitioner per service (public sector, primary care)	Full-time equivalent (FTE)	0.0000252604 (MD), 0.0001388889 (NS), 0.0000063368 (PY), 0.0000025347 (MT), 0.0000757813 (PH)	The Permanent Secretary Office, MoPH and Expert opinions from GMB sessions
Practitioner per service (public sector, acute care)	Full-time equivalent (FTE)	0.0100000000 (MD), 0.0100000000 (NS), 0.0007118056 (PY), 0.0002847222 (MT), 0.0002372685 (PT)	The Permanent Secretary Office, MoPH and Expert opinions from GMB sessions
Practitioner per service (public sector, emergency care)	Full-time equivalent (FTE)	0.0006076389 (MD), 0.0007638889 (NS), 0.0000781250 (PY), 0.0000312500 (MT)	The Permanent Secretary Office, MoPH and Expert opinions from GMB sessions
Practitioner per service (public sector, intermediate care)	Full-time equivalent (FTE)	0.0000781250 (MD), 0.0003125000 (NS), 0.0015625000 (PT), 0.0003125000 (PH)	The Permanent Secretary Office, MoPH and Expert opinions from GMB sessions
Practitioner per service (public sector, palliative and end-of-life care)	Full-time equivalent (FTE)	0.0001562500 (MD), 0.0007812500 (NS), 0.0003125000 (PY), 0.0003125000 (PT), 0.0007812500 (PH)	The Permanent Secretary Office, MoPH and Expert opinions from GMB sessions
Practitioner per service (public sector, long-term care)	Full-time equivalent (FTE)	0.0002604167 (MD), 0.0007812500 (NS), 0.0002604167 (NS), 0.0007812500 (PT), 0.0007812500 (PH)	The Permanent Secretary Office, MoPH and Expert opinions from GMB sessions
Practitioner per service (public sector, ambulatory care)	Full-time equivalent (FTE)	0.0000833333 (MD), 0.0001458333 (NS), 0.0000625000 (PT), 0.0000250000 (PH), 0.0000138889 (CPsy)	The Permanent Secretary Office, MoPH and Expert opinions from GMB sessions
Services capacity per team (public sector)	Episode/team/year	32,850 (primary care), 570 (acute care), 1947 (emergency care), 730 (intermediate, long-term care, palliative care), 18,250 (ambulatory care)	The Permanent Secretary Office, MoPH and Expert opinions from GMB sessions
Waiting time before leaving the profession	Year	3	The Permanent Secretary Office, MoPH and Expert opinions from GMB sessions
Healthcare team expansion ratio	Dimensionless	0–0.02	The Permanent Secretary Office, MoPH and Expert opinions from GMB sessions
Time allocation for administrative (not-patient care) work	Team expansion ratio	0.15–0.30	The Permanent Secretary Office, MoPH and Expert opinions from GMB sessions
*Healthcare education and labor market module*			
The capacity of HRH training program	Person/year	2800 (MD), 10,000 (NS), 1900 (PY), 1100 (MT), 950 (PT), 12,258 (PH), 200 (CPsy)	The Permanent Secretary Office, MoPH and Expert opinions from GMB sessions
Batch dropout ratio	Person/batch	0.03–0.20	Database of each professional council and Expert opinions from GMB sessions
Study period	Year	6 (MD), 5 (PY), 4 (NS, MT, PT, PH, CPsy)	Office of the Higher Education Commission, Ministry of Education
Workforce pool (not practicing, or working in other industries)	Person	672 (MD), 4545 (NS), PY (15,944), 952 (MT), 4569 (PT), 14,570 (PH), 0 (CPsy)	Expert opinions from GMB sessions

### Model parameters

The parameters used in our model are shown on Table 2. These parameters were used in the initial steady state of our model, which represents a dynamic equilibrium and is numerically sensitive to model parameters.

To test for policies, we evaluate the policies on four outcomes that concern health workforce planning at the national level. From our GMB process, the sufficiency of the health workforce in Thailand can be seen by (1) population health status, (2) unmet health needs, and (3) healthcare expenditures.

The first outcome is the overall population health status represented by the percentage of a healthy population in the country, which indicates an adequate health workforce in the effective healthcare delivery models for the demands of population health. Another population health outcome is the health-related quality of life (HRQoL) of the Thai population, which captures the degree and effectiveness of long-term care and palliative care necessary for aging, disabled, and terminal stage patients who cannot be converted to a healthy state. The second outcome is unmet health needs, which reflect limited access to necessary care for their health status. An inadequate health workforce does not only compromise population health status, but can also create long-waiting time, congested patients at healthcare facilities, and equitable access to necessary care. The third outcome is the healthcare expenditure, which is the primary concern of the government and partially address the cost-effectiveness of policy interventions from the societal perspective.

### Policy experimentation

We ran our system dynamics simulations under four scenarios. Model parameters were changed (i.e., service gap, out-of-pocket cost, and the number of doctors) to conduct policy experimentation and illustrate the potential impacts of each policy in the next 20 years (2017–2038) under the following scenarios:

1. *Scenario I business-as-usual (BAU):* All key policy variables were kept constant. Under this scenario, all model inputs, including the effectiveness of the available health workforce actively working in all healthcare delivery models in Thailand, was assumed to be equal and remain unchanged over the simulation time.
2. *Scenario II decentralizing primary care (Policy#1):* The health workforce planning takes into the account of decentralization of primary care units from the MoPH of the central government to the ownership of local governments, and also limiting new recruitments of physicians into the public facilities of from the year 2027 on.
3. *Scenario III expansion of public financing and modernizing primary care (Policy#2):* The health workforce planning takes into the account of expanding the public funding to care delivery by the private sector and also the modernization and digitalization of MoPH primary care units.
4. *Scenario IV major reforms of care delivery models (Policy#3):* The health workforce planning considers the significant reforms of all care delivery models by MoPH healthcare facilities. This scenario mainly shifts the focus from only filling the health workforce in hospitals care to produce a significant proportion of the health workforce that is better qualified for working in non-hospital settings.

### Model validation

The model is validated using unit consistency test, structural validity test, and behavioural replication test [18]. To test for unit consistency, we used the unit test function in the Stella Architect software. We focused on two dimensions. First, the unit of each variable must have the meaning and consistent with the description of that variable. The second dimension is that the unit must be consistent throughout the model. After testing for the unit consistency, the unit of all variables represents the real meaning of those variables. Besides, Stella software shows no unit error, which indicates that the unit is consistent throughout the model. Therefore, the model passes the unit consistency test.

For the structural validity test, we tested the model in the GMB sessions by showing the model to the group of experts who works in the healthcare industry, health systems researchers, and the government agencies who manage healthcare security and healthcare services. The experts agree that the structure of the model reflects the actual situation. Therefore, the model passes the structural validity test.

Lastly, we did a behavioural replication test. The reference model was drawn using multiple data, including the number of Thai populations by ages and their reported health state from the National Statistics Office’s Health and Welfare Survey 2007, 2009, 2011, 2013, and 2015. The number of the health workforce in Thailand by each type of care model was obtained by the researcher’s primary survey in December 2017 and January 2018. The silmulation results in the model can trace the actual numbers of Thai populations receiving care over time. Therefore, the model passes the behaviour replication test.

## Results

**Fig. 5:**
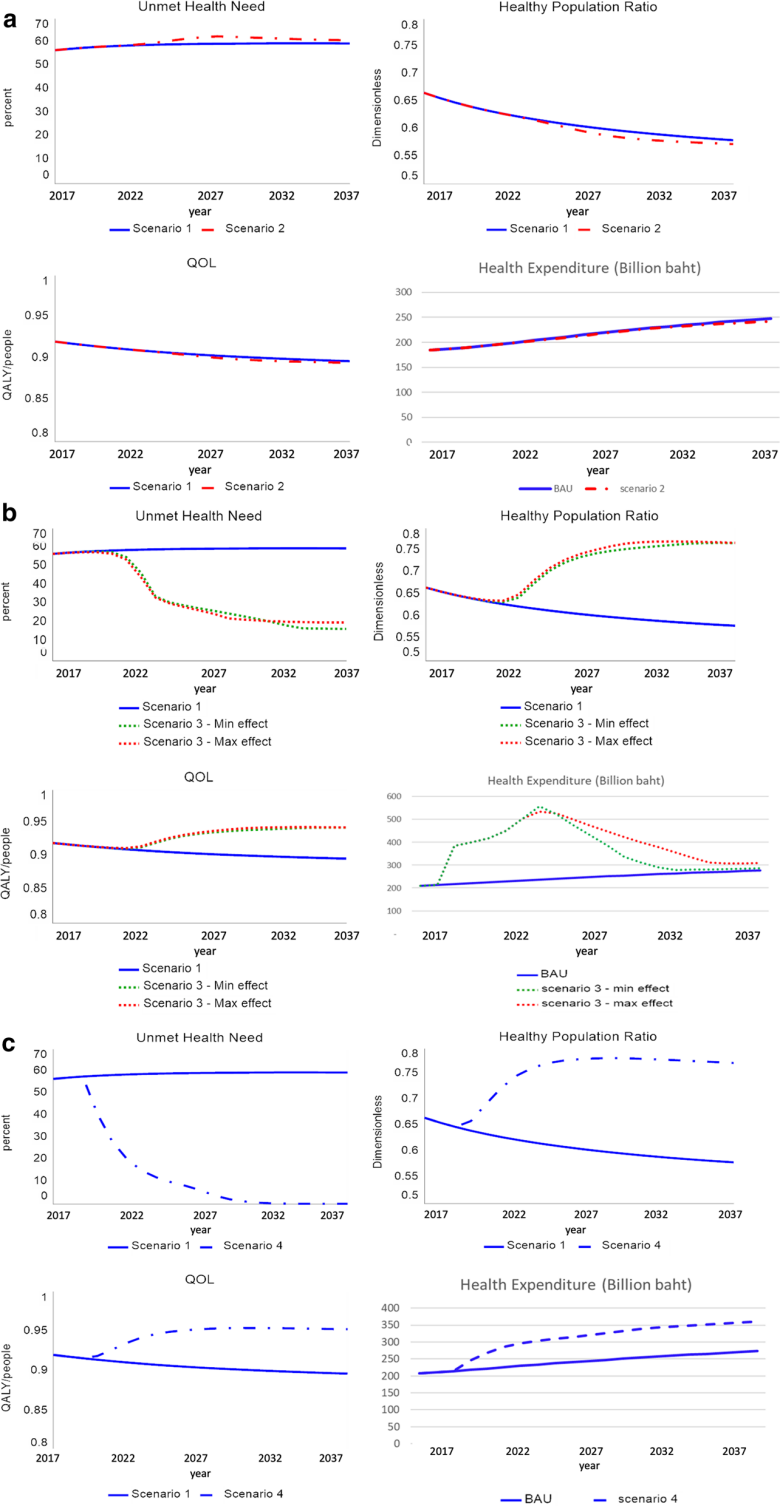
**a** Impacts of “decentralizing primary care” (Scenario II) on the health systems performance compared to the business-as-usual (Scenario I: BAU). **b** Impacts of “expansion of public financing and modernizing primary care” (Scenario III) on the health systems performance compared to the business-as-usual (Scenario I: BAU). **c** Impacts of “major reforms of MoPH care delivery models” (Scenario IV) on the health systems performance compared to the business-as-usual (Scenario I: BAU)

Our simulation modelling produced results, as shown in Fig. 5a–c, displaying the impacts of the four scenarios on the four primary outcomes. The three policy options were compared to our baseline or the “business-as-usual” (BAU) scenario. We can observe the consequences of current health workforce policies that most workforce have been working in hospitals.

Under Scenario I (the BAU), the population health outcomes, both the ratio of a healthy population and health-related quality of life, gradually got worse over the next two decades. Both health systems’ performance also declined, as the unmet health needs slowly increased, and healthcare expenditures kept rising over the whole period.

Under Scenario II (Policy#1), we considered the impacts of decentralization of primary care units from central government to local governments and limiting new recruitments of physicians into the MOPH facilities from the year 2027 on. These policy options emerged from our GMB process, but our simulation revealed that it produced almost the same patterns of systems behaviours like that of the BAU Scenario. The healthy population and unmet health needs of the people got slightly worse than that of the BAU approximately after 10 years of this policy implementation, or from the year 2027 on.

Under Scenario III (Policy#2), we considered the impacts of expanding public financing for private healthcare delivery while modernizing primary care in the public sector, especially implementing the digitalization of MoPH primary care units. From the year 2022 or approximately after 5 years of this policy implementation, the ratio of the healthy population and health-related quality of life rapidly improved. The unmet health also needs shapely dropped around the year 2022 and more gradually dropped furthermore after 2025. The simulation of total healthcare expenditures displayed an interesting pattern of “worse before better” by immediately and sharply increased after policy implementation but decreased approximately after eight years, or from the year 2025.

Lastly, under Scenario IV (Policy#3), we considered the impacts of significant reforms of all care delivery models by shifting the focus from only filling the health workforce in MoPH hospitals care and producing a substantial proportion of health workforce to promote non-hospital care. The ratio of a healthy population, health-related quality of life, and the unmet health need rapidly improved, similar to the pattern observed under Scenario III. However, we can observe the improvement slightly faster than that of Scenario III. The significant difference was on healthcare expenditures, which slightly increased from that of the BAU Scenario but not as highly increased as Scenario III. However, unlike Scenario III, healthcare expenditures never went down under Scenario IV.

## Discussion and conclusion

Our study was among the first to investigate plausible scenarios of the strategic health workforce planning by taken into the account of healthcare delivery reforms of either Thailand or other low-and middle- income countries (LMICs). The evidence can inform the governance of Thailand’s UHC in the next decades to come. Using the GMB process, the policymakers and stakeholders gained a better understanding of causal relationships among factors in Thai healthcare systems related to the sufficiency of the health workforce or the mismatch of supplies and demands of the health workforce. Moreover, and policy options were tested by our quantitative simulation modelling to compare the consequences of each policy.

As a significant proportion of primary care, long-term care, intermediate care, palliative care and end-of-life care in Thailand have been delivered in the hospitals, these care delivery models have been sharing the same health workforce with the acute care delivery systems. We potentially can redesign such care delivery systems to provide care outside the hospitals effectively. Without the changes of care models toward delivering non-hospital care models in non-hospital settings, the policy options for the national health workforce planning would rather be limited. Initiating significant reforms of all care delivery models, by shifting the focus from only filling health workforce hospitals care to promoting health workforce placements in non-hospital care settings, or creating new care delivery systems for the integration of hospital care and non-hospital, can lead to the most desirable outcome consistently with suggestions from a stream of literature on integrated care [19, 20] and value-based care [21, 22]. Primary care, among other non-hospital care models, can positively impact self-care of chronically ill patients, as evidence shows that primary care can improve the population's health literacy and self-management competencies [23, 24], a synergistic effect with public health services to reduce the unmet health needs furthermore.

Outstanding results of care redesign is observed by our SD model, especially when compared to merely hiring a new health workforce in the existing care models as depicted by the business-as-usual. The healthcare expenditures would increase by approximately 1.3 times of the starting year of 2017. More importantly, the better ratio of health population and the lower level of unmet health needs would result in fewer demands for the health workforce in the long run. The reduced unmet health needs can affect fewer demands for new facilities in both the public and private sectors. Overall, linking workforce planning strategy with healthcare delivery reforms would provide better outcomes in population health status and health systems performance. It would be a far superior policy option, especially when compared to implementing a set of new health workforce policies in the exiting healthcare delivery models. While the complexity of managing the health workforce can be significantly increased during healthcare reforms, at the same time, inadequate preparation of human resources for incoming health systems reforms also can impact the performance of health systems negatively [25].

Alternatively, policymakers can implement new health workforce policies that emphasize new financing mechanisms for existing care delivery models. The argument would be to increase the efficiency of the health workforce, their healthcare teams, and healthcare organizations. On the downside, as demonstrated by Scenario II, the unmet health needs of people without any access to necessary care would be kept at 20% in the next two decades. Yet, the healthcare expenditure would be approximately twofold in the first 8 years and then decreased to a similar level of the BAU Scenario, even with the assumption of using more information and communications technologies (ICTs) to receive a greater efficiency of health workforce utilization and care delivery models. While some policymakers believe using more ICT in healthcare delivery can be more efficient than producing and managing the health workforce, our findings are consistent with a stream of literature that suggests the limited effects of ICT without shifting resources among care models or improving the design of healthcare delivery systems [26–28]. Hence, training a new workforce or retraining the existing ones already working in the health systems would provide a much better outcome.

Beyond the healthcare expenditures, any potential policies that rely on the new workforce, payment mechanisms, or ICT systems implemented upon the existing healthcare delivery but not providing an incentive for the reforms of healthcare delivery will minimally affect the health status of the populations. Hence, health workforce policies with a focus on the reforms of healthcare delivery itself, e.g., one that promotes a more balance between hospital care and non-hospital care or a greater integration among care delivery models, should be preferred. However, these policy options are unlikely successful if only a limited number of healthcare providers in the market offer an integrated care. To increase the supplies, the focus of health workforce policies should not limit only public providers and include both public and private providers who qualified. For instance, primary care clinics or rehabilitation centers in the private sector are currently not a major focus of the reimbursement systems of all major public healthcare funds. In Thailand, the payer, such as UCS, can team up with public or private hospitals to establish an integrated care process for their patients. However, by this option, healthcare expenditures can increase more rapidly in the early years due to the higher unit cost of healthcare services in the private sector compared to that of public providers.

Although our simulation shows that significant care delivery reforms nationwide can be more effective, it could be less feasible in the short-run. The high cost of substantial healthcare delivery reforms is one of the unintended consequences creating systems inertia or policy resistance. In contrast, limiting healthcare delivery reforms to modernizing primary care could be more feasible for implementation in the short run. Expanding the public funding to cover care delivered by the private sector could be less feasible only if perceived as downplaying public facilities' roles. If so, resistance from MoPH could be expected, as MoPH traditionally plays both functions of the policymakers (the planner of the national workforce) and the provider (the owner of health care organizations providing health care services to most Thai populations). Lastly, suppose policymakers are not motivated to develop new professional care teams (e.g. family medicine providers), or too attached to the legacy of using non-professional workers in primary care (e.g. community health volunteers), MoPH may not be able to modernize its primary care as proposed. Evidence also suggests that, with the increasing urbanization of rural villages in Thailand, the volunteers no longer serves as the point of entry into the healthcare systems as they had successfully been in past decades [29].

As put forth by Milstei et al. [30], system dynamics modelling can demonstrate the consequences of policy options of healthcare reforms in a more comprehensive way. However, our study may have some limitations in predicting future outcomes if the assumptions used to construct our system dynamics modelling is too far from the complex reality. The health outcomes can be altered from the simulated ones for several reasons, including (1) the quantity and quality of health workforce in the future might be inadequate for unexpectedly rising health demands of Thai populations, (2) the patients might have a different preference for specific types of healthcare teams, or (3) their accessibility to new care models might not be as high as expected. Moreover, the healthcare expenditures may increase even more than the simulated numbers if the government expands the UHC benefit packages from the existing ones. Lastly, due to the exploratory nature of our study, our model reveals the trend of population health status and systems performance outcomes as the consequences of each policy option. Still, we did not aim to precisely forecast an exact amount of healthcare expenditures or any other results. More specifically, for simulated healthcare expenditures, we did not take into account of the inflation in our model yet.

In future studies, researchers can use SD models in at least two directions to support the policy decision process on national health workforce planning. First, researchers may identify emerging trends that potentially impact the health workforce's demands and supplies, and propose new policy options that address them. For instance, the massive demands for COVID-19 vaccination by both vulnerable and general populations and rapid adoption of digital health solutions in care delivery models during the COVID-19 pandemic could impact the demands and the supplies of the national health workforce in short and long terms. Simulating such updated policy options would keep the modelling relevant to the current policy process. Second, our study aims to capture the big picture of the health workforce planning, but national policymakers may also need a policy decision support tool for a more specific planning issue. For instance, provided a total number of physicians needed in our future health systems, policymakers may also want to learn more about the appropriate ratio between general practitioners and medical specialists, or the right number of each clinical speciality. To answer such questions, another modelling exercise with a narrower scope would be helpful for policy decision-making.

Building upon the present study, policymakers of healthcare reforms can benefit from further analyses. The synthesis of additional policy options by group model building and testing such policies by simulation modelling can help not only the strategic planning health workforce at the national level but also the planning and evaluation of the ongoing UHC reforms. Our modelling process also informs policymakers and stakeholders about what data in health information systems is crucial to the strengthening of UHC governance, particularly regarding managing the health workforce and health systems performance. Hence, this iterative nature of data collection and data analysis could be a lesson learned for the UHC policy process, not only in Thailand but also in other LMICs.

## Data Availability

The datasets used and analyzed during the current study are available from the corresponding author on reasonable request.

## Abbreviations

HRH: Human resource for health
GMB: Group model building
CLD: Causal loop diagram
SFD: Stock and flow diagram
HP: Healthy population
SP: Population with simple illnesses
CP: Population with complex illnesses
MD: Medical doctor
NS: Nurse
PY: Pharmacist
MT: Medical technologist or medical laboratory technologist
PT: Physical therapist or physiotherapist
PH: Public health officer
CPsy: Clinical psychologist
FTE: Full-time equivalent
HRQoL: Health-related quality of life
BUA: Business-as-usual
MoPH: The Ministry of Public Health
LMICs: Low-and middle-income countries
ICT: Information and communications technologies
