# Correction to: System dynamics modelling of health workforce planning to address future challenges of Thailand’s Universal Health Coverage

**DOI:** 10.1186/s12960-021-00589-w

**Published:** 2021-04-01

**Authors:** Borwornsom Leerapan, Pard Teekasap, Nipaporn Urwannachotima, Wararat Jaichuen, Kwanpracha Chiangchaisakulthai, Khunjira Udomaksorn, Aronrag Meeyai, Thinakorn Noree, Krisada Sawaengdee

**Affiliations:** 1 Faculty of Medicine Ramathibodi Hospital, Mahidol University, 270 Rama VI Road, Ratchathewi, Bangkok, Thailand; 2 Faculty of Business Administration, Stamford International University, Bangkok, Thailand; 3 Faculty of Dentistry, Chulalongkorn University, Bangkok, Thailand; 4 International Health Policy Program, Thailand (IHPP), Ministry of Public Health, Nonthaburi, Thailand; 5 Office of the Permanent Secretary, Ministry of Public Health, Nonthaburi, Thailand; 6 Faculty of Pharmacy, Prince of Songkla University, Songkla, Thailand; 7 Faculty of Public Health, Mahidol University, Bangkok, Thailand; 8 Oxford Centre for Global Health Research, Nuffield Department of Medicine, University of Oxford, Oxford, UK

## Correction to: Hum Resour Health (2021) 19:31 https://doi.org/10.1186/s12960-021-00572-5

Following the publication of the original article [1], an error was identified in the Conclusions of the Abstract section.

The updated conclusion is given below and the change has been highlighted in **bold typeface**.

The sentences currently read:

Our SD modelling confirmed that shifting the care models to address the changing health demands can be a high-leverage policy of health workforce planning, although very difficult to implement in the short term. **of health workforce planning, although very difficult to implement in the short term**.

The sentence should read:

Our SD modelling confirmed that shifting the care models to address the changing health demands can be a high-leverage policy of health workforce planning, although very difficult to implement in the short term.

The original article [1] has been corrected.
